# Non‐Native Woody Plant Species Show Different Leaf Functional Traits and Herbivory Levels From Native Ones in the Urban Areas of Beijing, China

**DOI:** 10.1002/ece3.71947

**Published:** 2025-08-08

**Authors:** Yingjie Wang, Shuang Zhang, Xingwu Duan, Keming Ma

**Affiliations:** ^1^ State Key Laboratory of Urban and Regional Ecology, Research Center for Eco‐Environmental Sciences Chinese Academy of Sciences Beijing China; ^2^ Institute of International Rivers and Eco‐Security Yunnan University Kunming China

**Keywords:** herbivory, leaf functional traits, non‐native plants, phylogenetic history, urban ecology, urbanization

## Abstract

A large number of non‐native species have been introduced to urban ecosystems, and it is a distinctive feature of the urbanization process. However, it is unclear whether these non‐native species have similar functional traits to native ones and are similarly integrated into the local food web. We evaluated the differences in leaf functional traits and herbivory between native and non‐native species of common woody plants in 50 parks in Beijing, China. The nutrient contents, defensive traits, and levels of herbivory were measured in 2681 leaves across 138 (52 native and 86 non‐native species) woody plant species. Results show that compared to native species, non‐native trees showed greater potential for short‐term carbon sequestration, lower nutrient contents, and chemical defense but similar levels of herbivory. Non‐native shrubs had lower carbon contents and herbivory levels than native shrubs. Phylogenetic history explained much more of the variance in plant traits and herbivory than spatial variation, suggesting the high homogeneity of environments among different urban parks. Furthermore, the variation in leaf traits and herbivory of non‐native species had higher uncertainty than that of native species. Our research findings indicate that compared to native species in urban ecosystems, non‐native species have reduced plant–herbivore energy flow to primary consumers, which may hinder biodiversity at higher nutrient levels. In the future, urban parks should incorporate more native plant species and enhance environmental heterogeneity.

## Graphical abstract

Non‐native plants differ in traits but show similar herbivory to native species. Phylogenetic history drives trait variance more than spatial factors in urban parks. Native plants boost biodiversity; some non‐natives enhance urban carbon storage.

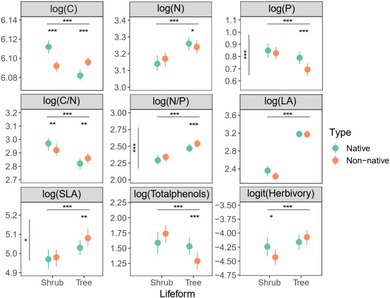

## 1 Introduction

With the intensified urbanization process, a large number of non‐native plants have been introduced to cities, leading to the popularity of non‐native species in urban ecosystems (Threlfall et al. 2016). While these species enhance urban greening, they may alter leaf functional traits and herbivory dynamics, impacting biodiversity and ecosystem functions. However, it is unclear whether these non‐native species have similar functional traits and ecological effects as native ones (Leffler et al. 2014; Hulme and Bernard‐Verdier 2018). This is a fundamental question that must be addressed in urban ecology.

Native and non‐native plants in cities may have different functional properties (Ordonez 2014), leading to different ecological effects. The proportion of non‐native species in urban ecosystems is higher compared to natural ecosystems (Threlfall et al. 2016; Tartaglia and Aronson 2024). However, these non‐native species are mostly introduced by humans based on landscape, aesthetic, and other value considerations (Pyšek et al. 2015; Lai et al. 2019; Mata et al. 2020; Bayón et al. 2021), and we do not have a clear understanding of their ecological properties and functions (Schwarz et al. 2017). By altering community structure (Seastedt and Pysek 2011; Santos et al. 2016; Bardgett 2017; de Souza e Silva et al. 2020) and functional traits (Smith and Knapp 2001; Leffler et al. 2014; Sandel and Low 2019), non‐native plants can profoundly influence key ecological processes, ranging from ecosystem‐level functions like nitrogen cycling (Castro‐Diez et al. 2014) and resource dynamics to the reorganization of soil microbial communities and food webs (Seastedt and Pysek 2011). However, it is difficult to generalize the differences in leaf functional traits between native and non‐native plants in urban ecosystems based on current studies (Leffler et al. 2014). In natural or experimental settings, successful invaders are often characterized by a fast resource‐use strategy, enabling rapid growth (Burns 2006) through traits like higher specific leaf area (SLA)—a trait reflecting both rapid light capture and lower physical defense—and photosynthetic rates (Pattison et al. 1998; Gulias et al. 2003; Lake and Leishman 2004; Burns 2006; Yu et al. 2020), coupled with a high‐nutrient strategy reflected in elevated leaf nutrient concentrations (Craine and Lee 2003; Burns 2006; Leishman et al. 2007; Montesinos 2022). Nevertheless, non‐native species do not always conform to this paradigm, with some studies finding atypical trait combinations such as lower photosynthesis co‐occurring with shorter leaf lifespan (Durand and Goldstein 2001), and others reporting no significant differences in SLA and leaf nutrient concentrations (Liu et al. 2017). The uncertainty regarding these trait differences is even more pronounced in urban ecosystems. The few available urban studies present conflicting evidence on plant resource‐use strategies. For instance, SLA—a key indicator of resource acquisition efficiency—shows contradictory patterns, with some studies reporting lower SLA (Zeeman et al. 2018) and higher (Liñán‐Vigo and Núñez‐Farfán 2024) in non‐native species. At the same time, other urban studies consistently report that non‐native species possess higher carbon stocks (Khan et al. 2020), higher leaf nutrient contents (Heberling and Fridley 2013) and faster resource acquisition ability in urban areas (Heberling and Fridley 2013; Díaz de León Guerrero et al. 2020). This striking inconsistency in leaf functional traits reveals a critical knowledge gap in urban invasion ecology, highlighting that current generalizations are limited and underscoring the urgent need for comprehensive, multi‐species studies in these unique ecosystems, where species introduction is not a natural process. Therefore, the differences in the functional attributes of native and non‐native species in the city are currently not clear, nor are their effects on ecological processes. Alongside these leaf functional traits, a critical aspect of ecological strategy is its interaction with herbivores. Herbivory is one of the most common and important ecological processes that determine the growth, reproduction, survival, and even the maintenance of biodiversity in plants (Kozlov and Zvereva 2017). Fewer primitive studies showed that non‐native plants have lower levels of herbivory in urban ecosystems than native plants, but all these studies were conducted with a limited number of plant species (with a maximum of 23 species) (Matter et al. 2012; Frank 2014; Grunzweig et al. 2015; Parsons et al. 2020); therefore, whether this is a general pattern is still an open question. According to the prediction of the enemy release hypothesis (ERH) (Keane and Crawley 2002; Colautti et al. 2004; Liu and Stiling 2006), non‐native species should have lower levels of herbivory because of the lower abundance of their enemies in the introduced area. However, although the hypothesis has been supported by some studies in natural ecosystems (White et al. 2008; Prior et al. 2015), its generality in managed and novel urban ecosystems remains understudied and has rarely been tested. The evolution of increased competitive ability (EICA) hypothesis states that non‐native plants should reduce investments in resistance to herbivores and increase investments in nutrient acquisition, growth, and reproduction (Blossey and Notzold 1995; Callaway and Ridenour 2004; Callaway et al. 2022). Further research is needed to unravel how these dynamics play out in the unique contexts of urban ecosystems, where ecological interactions are heavily influenced.

Since native species have a long‐term adaptive evolution to the local environments (Occhipinti 2013; War et al. 2018), their variation in leaf functional traits and herbivory should be largely governed by their phylogenetic history. In contrast, non‐native species lack long‐term adaptation to the local environments (Tallamy et al. 2010; Fickenscher et al. 2014; Mata et al. 2021), and the variations in their functional traits and herbivory might be more susceptible to the environmental factors rather than their phylogenetic histories. Both hypotheses have been explicitly tested in natural communities (White et al. 2008; Bossdorf 2013; Prior et al. 2015) but rarely in cities (Padullés Cubino et al. 2021). The relative contribution of phylogenetic history versus current local environments to the variation in plant function has not been evaluated in urban ecosystems. Concurrently, diverse life forms of woody plants, such as trees and shrubs, exhibit varied responses to urban alterations, encompassing modifications in resource acquisition, defensive capabilities (Xiao et al. 2021), and the balancing strategies of leaf traits (Xu et al. 2023). Hence, it is imperative to investigate the influence of these life forms.

In this study, we compared the nutritional, defensive traits, and herbivory of leaves in native and non‐native woody plant species in urban parks in Beijing, China. Specifically, we predict that in urban ecosystems, (1) compared to native species, non‐native plant species should show lower levels of herbivory and defensive levels but higher nutrient contents in their leaves, with this pattern potentially varying between life forms; (2) the relative contribution of phylogenetic history to the variation in leaf traits and herbivory should be higher in native species.

## 2 Materials and Methods

### 2.1 Study Site

The study was conducted in Beijing, China (39°28′–41°05′ N, 115°25′–117°30′ E). It has a north temperate semi‐humid continental monsoon climate with high temperatures and rain in summer and cold and dry in winter. The mean annual rainfall is 448 mm, and the mean annual temperature is 14°C. Beijing is one of the most rapidly urbanizing cities in China (Hu et al. 2018), which is densely populated and highly urbanized. According to official statistics, the population of Beijing at the end of 2021 is 21,890,000, and the percentage of the urban population is 87.50% (National Bureau of Statistics of China 2022).

### 2.2 Leaf Functional Traits and Herbivory Damage Measurements

**FIGURE 1:**
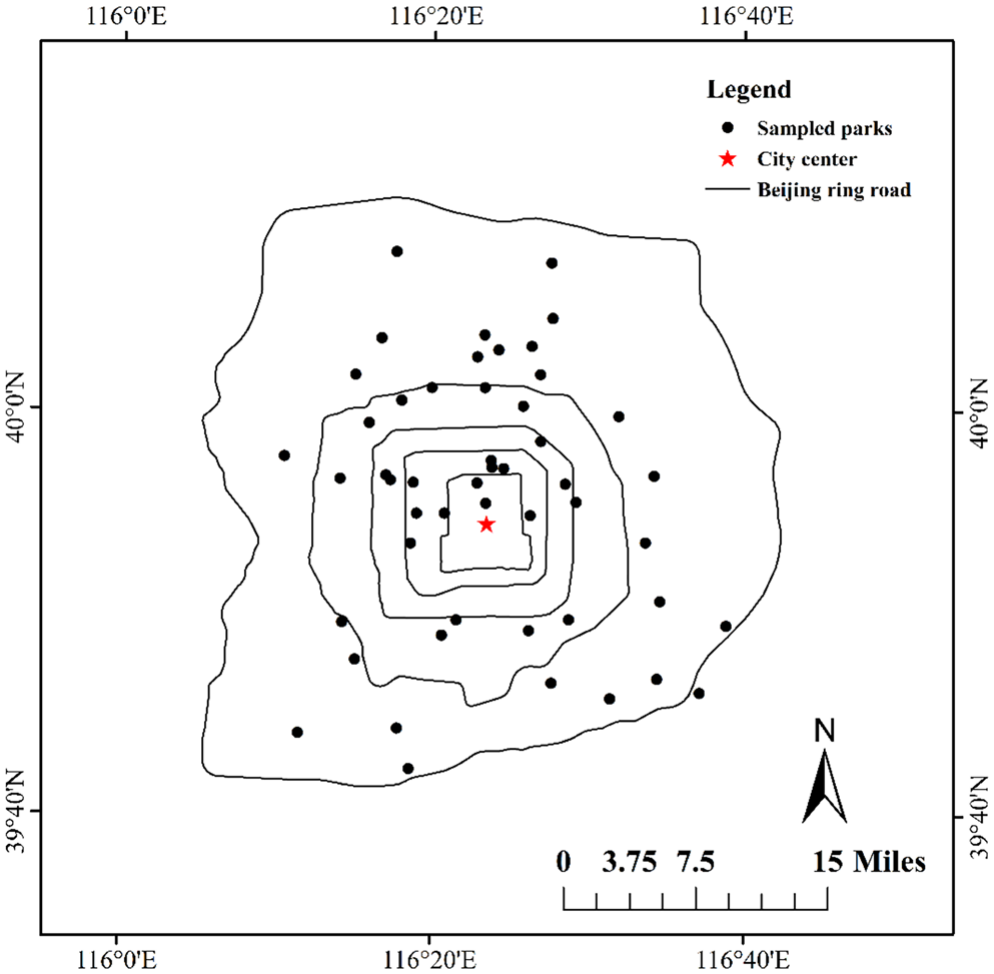
The study area and sampled parks. Eeach point represents a sampled urban park (*n* = 50).

Our study was conducted across 50 urban parks, about 4.69% of the total number of urban parks in Beijing. To ensure representativeness, our selection intentionally encompassed the full spectrum of common park types, from large, comprehensive, and historical parks to smaller community and pocket parks. All these parks are evenly distributed across administrative districts within the sixth ring road of Beijing to exclude the possible effects of elevational variation on the results (Figure 1). All the sampled leaves were collected from July to September 2021. This timing was deliberately chosen to ensure that the sampled leaves were fully expanded and at their peak physiological activity, thus standardizing the phenological stage across all species. In each park, we walked in the park along a circular path and collected the leaves of all woody plant species that were found along the transect. For each species, three or more individual plants were sampled. For species with less than three individuals that could be found, we sampled by the actual number of individuals. For each plant, we walked around and randomly collected 10 fully expanded leaves. Across all 50 parks, this resulted in a total of 2681 individuals from 138 species being sampled, with a mean of 19.4 individuals per species. Then, these leaves were photographed from a vertical leaf direction. To estimate leaf herbivory, we used the ZAX Herbivory Trainer‐Free software (Xirocostas et al. 2022) to quantify the percentage of leaf area removed, an approach chosen for its statistically robust balance between accuracy and the high throughput required for our large‐scale study. For each individual tree, 10 leaves were measured for herbivory. All estimations were performed by the same researcher (Shuang Zhang) to avoid estimation bias. The individual plant average value of herbivory was used in subsequent data analysis. We classified native and non‐native plants according to a newly published species list of vascular plants in Beijing (Xiao et al. 2022).

In total, eight leaf functional traits were measured, including leaf area (LA; cm^2^), specific leaf area (SLA; cm^2^/g), C (g/kg), N (g/kg), and P (g/kg) contents, total phenols contents (g/kg), C/N, and N/P. Leaf C, N, and P are indicators of nutrient content (Kobe et al. 2005; Hernández‐Montes et al. 2019), SLA is an indicator of leaf nutrient acquisition capacity, growth strategy as well as physical defense (Peeters 2002; Wright et al. 2004; Perez‐Harguindeguy et al. 2013). Leaf size is an indicator of leaf photosynthetic rate (Cary and Pittermann 2018), and total phenol is an indicator of leaf chemical defense (Mediavilla et al. 2018; Wang, Xiao, et al. 2021).

All samples were taken back to the laboratory, dried at 50°C for 48 h, and then sealed for storage in a cool and dry place. These adequately dried leaf samples were then used for leaf area and specific leaf area determination.

Leaf area (cm^2^) was measured using ImageJ image (version 1.53, National Institutes of Health, USA). SLA (cm^2^/g) was measured using a puncher (6 mm diameter) and an electronic balance with an accuracy of 0.1 mg. For each tree, we selected five relatively intact leaves and sampled four punches for each leaf. SLA was measured as the total area/total weight (in square centimeters per gram) of the 20 punched leaf segments (Zhang et al. 2023).

After testing leaf area and specific leaf area, the dried leaves of the same species from each park were combined and thoroughly crushed with a ball mill (retsch mm400) at a frequency of 30Hz for 30–60 s. Afterward, they were placed in sealed jars and stored in a dry, cool place for subsequent measurements.

Leaves of the same species from each park were combined and ground together for testing leaf nutrient and defense contents. We weighed 0.025 g of each sample for leaf carbon and nitrogen content, evaluating on an elemental analyzer (Element vario MAX cube, Germany). Leaf phosphorus content was measured on Inductively Coupled Plasma Mass Spectrometry (ICP‐MS) (Agilent 7500a, USA). Total phenols were measured by the Folin–Ciocalteu method (Waterman and Mole 1994), with gallic acid used as a standard.

### 2.3 Statistical Analysis

The nomenclature was cross‐referenced and standardized against major taxonomic databases, primarily the Plants of the World Online (POWO) and Flora of China (FOC). The coefficient of variation (CV) for each leaf functional trait and herbivory was calculated as the standard deviation divided by the mean, multiplied by 100 to express the result as a percentage. A mixed effects model was used to detect differences in leaf functional traits and herbivory between native and non‐native plant species. In the model, leaf functional traits or herbivory were set as the response variable, while plant type (native or non‐native), life form as well as their interactions were set as fixed variables. Park identity was set as the random effect to account for the non‐independence of data points from the same park. The mixed effects model was fitted using the “lmer()” function in the lme4 package (Bates et al. 2015). Post hoc pairwise comparisons of the estimated marginal means were conducted using the “emmeans” package (Lenth et al. 2025) to examine the main effects of type and life form, as well as their interaction contrasts.

To take into account the effects of phylogenetic history on the variance of leaf traits and herbivory, phylogenetic mixed effect models were used to explore the difference between native and non‐native plant species in leaf traits and herbivory. We constructed a phylogenetic tree that included all the collected species in our study using the “V.PhyloMaker” package in R (Jin and Qian 2019). After that, we transformed the phylogenetic tree to a correlation matrix with the “vcv.phylo()” function by setting cor = T in the “phytools” package (Revell 2012). Then the correlation matrix was included in the phylogenetic mixed effect model (Kubelka et al. 2018).

The phylogenetic mixed effects model was fitted using the “relmatLmer()” function in the lme4qtl package (Ziyatdinov et al. 2018). In the model, leaf functional traits or herbivory were set as the response variable, while life form was set as fixed variables. We included life form because of its strong effects on leaf traits and herbivory (Zhang et al. 2016). The correlation matrix was included in the model with the species name as the random effect. Park identity was also set as the random effects to account for the non‐independency of data points from the same park. The use of a phylogenetic mixed effects model was essential, particularly given the results of our pre‐analysis checks. To test for potential confounding between our fixed effect (life form) and phylogeny, we calculated Fritz & Purvis's *D* statistic to quantify the phylogenetic signal in the life form trait (*D* = 0.072, *p* < 0.001) using the “caper” package (Orme et al. 2023). This confirmed that our approach statistically controls for such phylogenetic structure via variance partitioning and was necessary to obtain unbiased estimates. Phylogenetic signal was estimated via variance partitioning, using the standard formula of Pagel's *λ* (Halliwell et al. 2025):

$$\lambda =\frac{\sigma_{\mathrm{phy}}^{2}}{\sigma_{\mathrm{phy}}^{2}+\sigma_{\mathrm{res}}^{2}}$$

In our model, $\sigma_{\mathrm{phy}}^{2}$ represents the variance explained by shared evolutionary history, while $\sigma_{\mathrm{res}}^{2}$ includes both spatial variation (e.g., park identity) and residual error, since these components were not modeled separately. We visualized the relative contribution of each component as a percentage of total variance to illustrate phylogenetic signal.

We log‐transformed data for leaf functional traits and logit(herbivory + 0.001) transformed data for herbivory to improve the normality of residuals as the logit transformation is considered more appropriate for proportional data than older methods like arcsine transformation (Warton and Hui 2011).

## 3 Results

**FIGURE 2:**
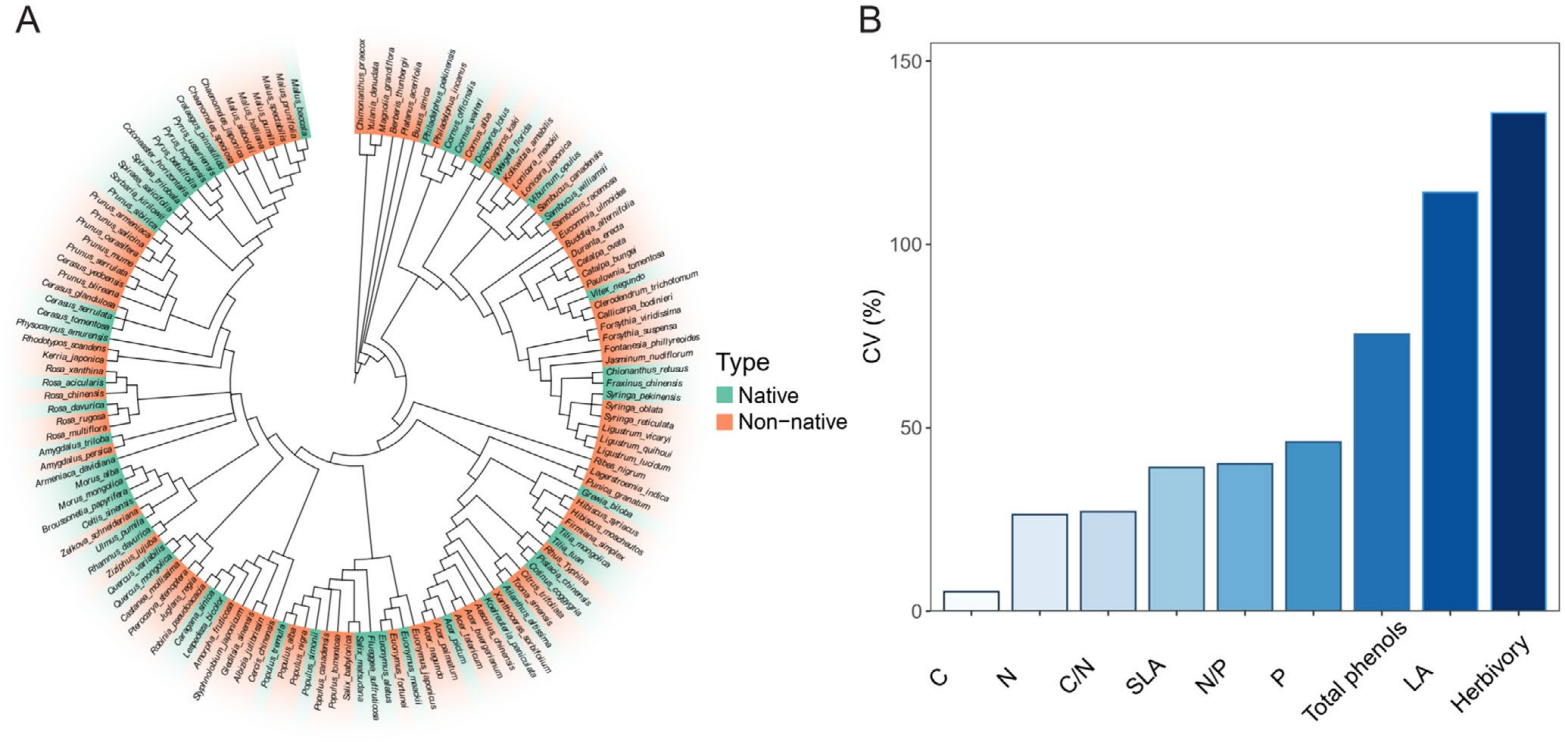
(A) The phylogenetic tree of the native and non‐native species used in this study. (B) Coefficient of variation of leaf functional traits and herbivory.

In total, we collected 26,165 leaves from 2682 individual plants belonging to 36 families, 83 genera, and 138 species (61 shrub species and 77 tree species). Of all the sampled species, 52 are native species, and 86 are non‐native species. The phylogenetic history of these species is shown in Figure 2A.

The mean level of herbivory was 2.4% (SE = 0.06%, *N* = 2682). The mean C content was 444.17 g/kg (SE = 0.46 g/kg, *N* = 2648), the mean N content was 25.54 g/kg (SE = 0.13 g/kg, *N* = 2648), the mean P content was 2.34 g/kg (SE = 0.21 g/kg, *N* = 2654), the mean C/N was 18.61 (SE = 0.10, *N* = 2648), the mean N/P was 12.54 (SE = 0.10, *N* = 2648), the mean LA was 26.48 cm^2^ (SE = 0.59 cm^2^, *N* = 2652), the mean SLA was 165.04 cm^2^/g (SE = 1.256 cm^2^/g, *N* = 2652), and the mean total phenol content was 6.29 g/kg (SE = 0.18 g/kg, *N* = 713) across different woody plants. The level of leaf herbivory showed higher variability than all other functional traits (Figure 2B).

### 3.1 Differences in Leaf Functional Traits and Herbivory Between Native and Non‐Native Plants

**FIGURE 3:**
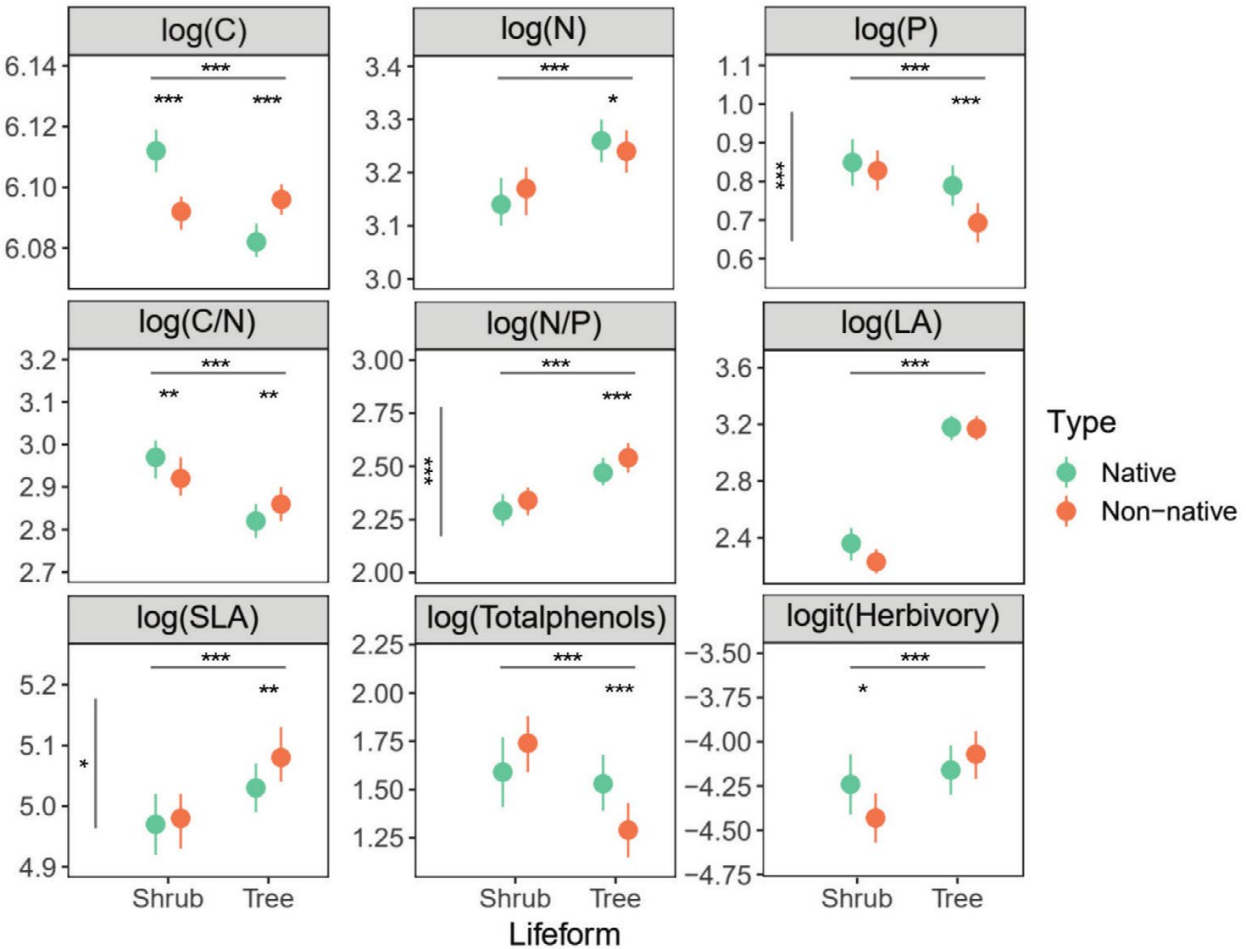
Comparison of leaf functional traits and Herbivory of native and non‐native plants (mean values and 95% CIs). The significance levels were indicated by asterisks (***, *p* < 0.001, **, *p* < 0.01, *, *p* < 0.05). The asterisks above the horizontal lines indicate significant differences in life form. The asterisks below the horizontal lines indicate significant differences in types within the same life form. The asterisks on the left indicate significant differences between native and non‐native species.

Both leaf traits and herbivory varied with plant life forms. Therefore, the data on trees and shrubs were analyzed separately. For trees, native species had higher leaf N and P contents and total phenol contents but lower SLA than non‐native species (Figure 3). However, native (mean = 1.44%, SE = 7.12%, *n* = 695) and non‐native (mean = 1.58%, SE = 6.95%, *n* = 828) tree species showed similar levels of leaf herbivory (see Tables S1–S4 for full statistical details). The percentage difference in mean herbivory between non‐native and native tree species was only 9.7% (Figure 3).

For shrubs, native species had higher leaf C contents, C/N, and herbivory levels than non‐native species (Figure 3). The level of herbivory in native shrubs (mean = 1.32%, SE = 8.66%, *n* = 345) was significantly higher than that in non‐native ones (mean = 1.08%, SE = 7.01%, *n* = 814).

### 3.2 The Relative Contribution of Phylogenetic History and Spatial Variation to the Variances of Leaf Functional Traits and Herbivory

**FIGURE 4:**
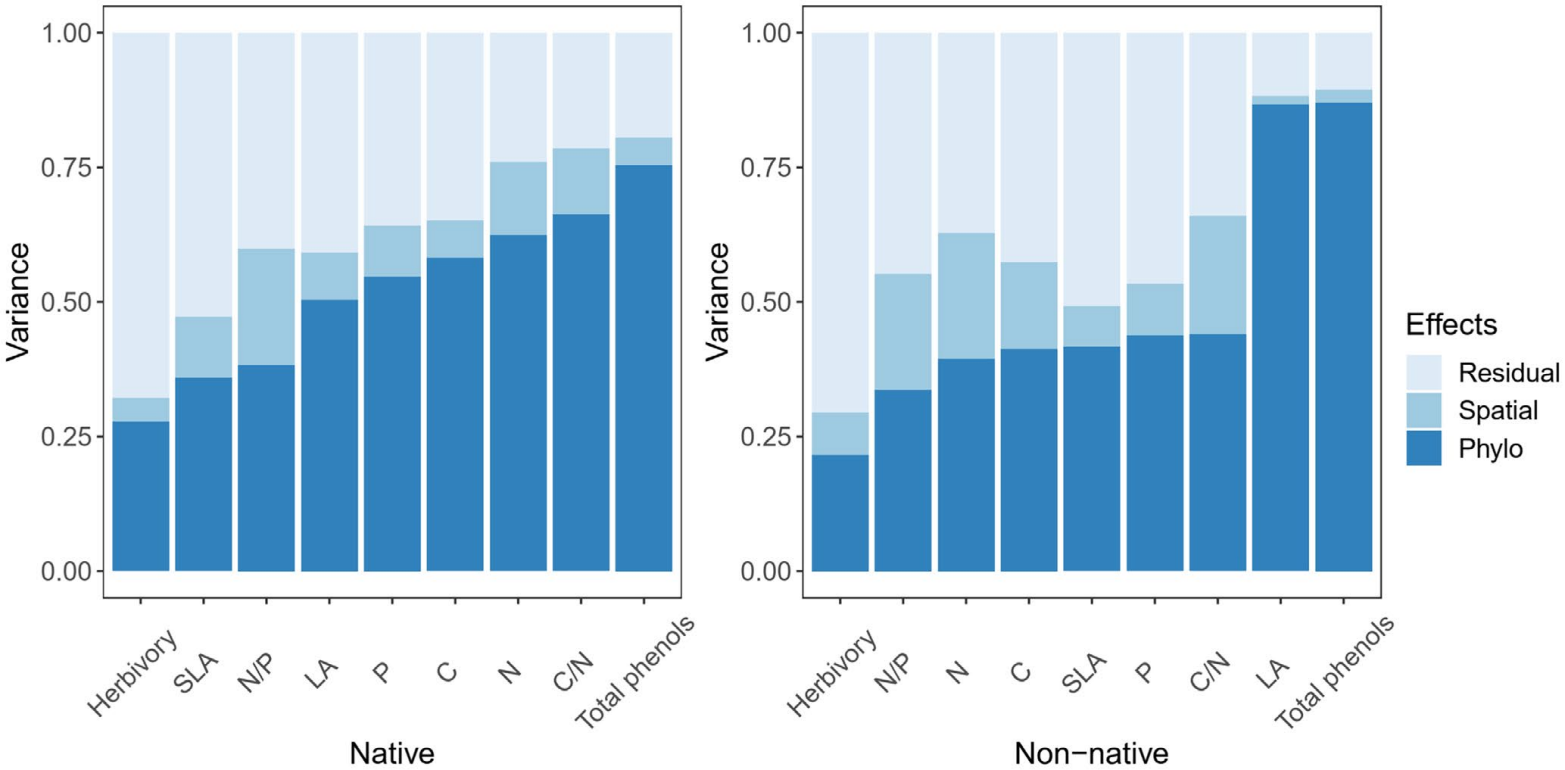
The contribution of phylogenetic history and spatial variation on the variances in leaf traits and herbivory between native and non‐native species.

In all the cases, phylogenetic history contributed more variance in leaf traits and herbivory than spatial variation, both in native and non‐native species (Figure 4). These contributions were estimated by variance partitioning, based on random effect variance components extracted from the mixed effects models—a necessary approach given the significant phylogenetic signal we detected in the life form trait (*D* = 0.072, *p* < 0.001). Compared to leaf functional traits, herbivory had the lowest proportion of variance (32.19% and 29.48%), which can be explained by phylogenetic history and spatial difference (Figure 4). In both native and non‐native species, phylogenetic history and spatial variation had the weakest effects on herbivory and the strongest effect on the total phenols content of leaves.

**FIGURE 5:**
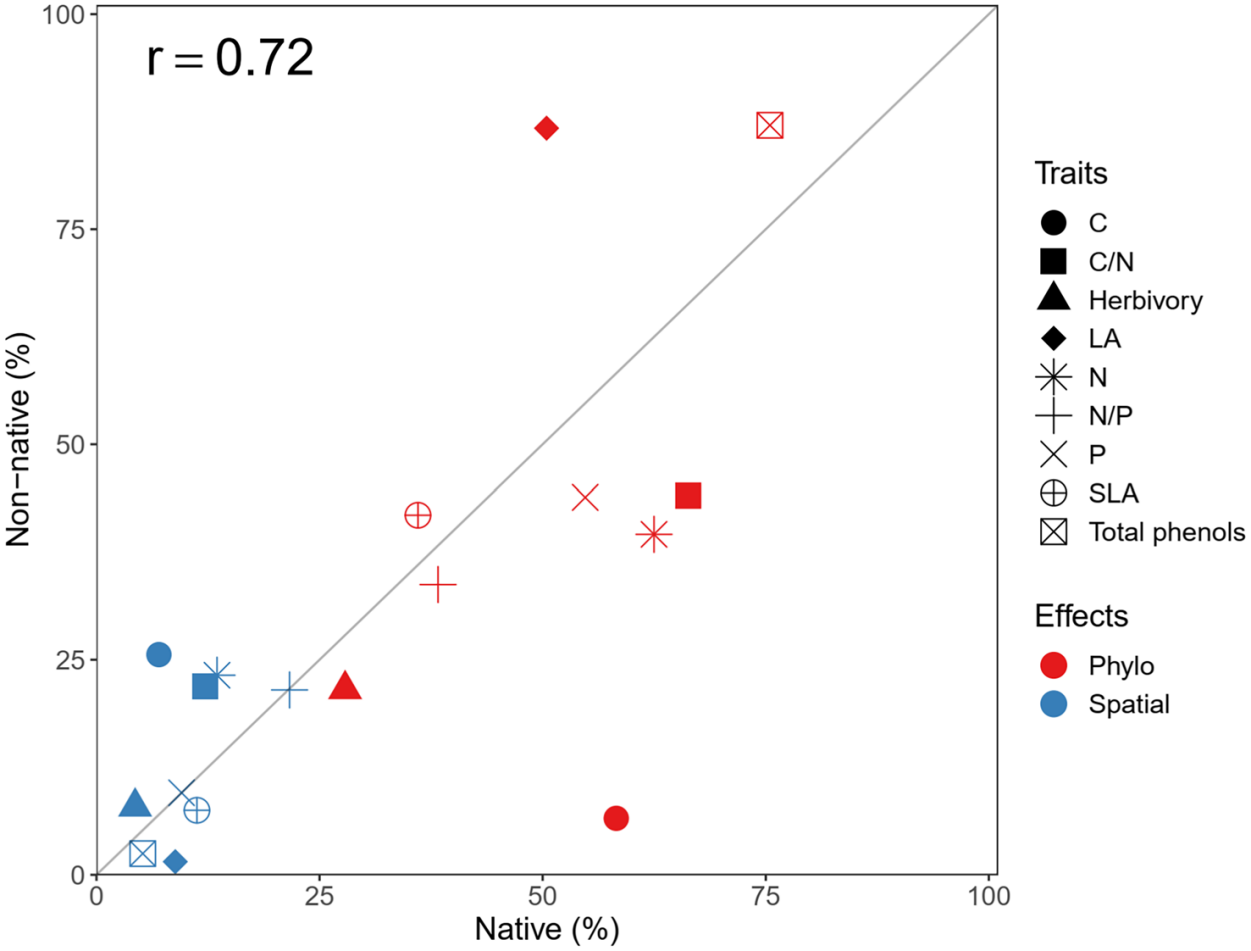
Correlation between the contribution of different factors (phylogenetical history and spatial variation) to the variances of leaf traits and herbivory. Colors represent the sources of variation. A 1:1 reference line was shown.

The contribution of phylogenetic history to variance in herbivory was higher in native species than in non‐native ones (27.86% vs. 21.57%), while the contribution of spatial variation to the variance in herbivory was about two times higher than the contribution of native ones (14.31% vs. 7.74%) (Figures 4 and 5).

The contribution of phylogenetic history and spatial differences to the variance in leaf traits and herbivory was positively correlated (*r* = 0.72) (Figure 5). Ecologically, deviations from the 1:1 line indicate differences in the relative influence of phylogenetic history or spatial variation on traits between native and non‐native species. However, phylogenetic history and spatial variation explained more variance in the measured variables in native species than in non‐native ones (There are 11 points under the 1:1 line and only 7 points above the line in Figure 5). This pattern indicates that the traits of non‐native species are more challenging to predict based on phylogenetic history and site‐specific environmental factors than those of native ones.

## 4 Discussion

In this study, through extensive sampling in a typical megacity with a high rate of urbanization, we show that non‐native tree species had lower physical defense, such as a significantly higher SLA, and also had lower leaf nutrient content compared to native ones. For shrubs, the level of herbivory was lower in non‐native species. Furthermore, the traits of non‐native species showed lower predictability from phylogenetic history and spatial location compared to those of native species, as a smaller proportion of their variance was explained by these factors in our models. These results suggest that the non‐native species have both different traits and functions from native ones in urban ecosystems.

Our study indicated that non‐native woody plants, as a group, have adopted a distinct resource‐use strategy compared to native ones. Non‐native woody plants have lower levels of leaf nutrient contents (e.g., lower P content), lower physical defense (e.g., higher SLA) and enhanced resource acquisition rates (e.g., higher SLA and N:P ratio), indicating a fast‐return strategy (Wright et al. 2004) optimized for greater phosphorus use efficiency. This allows these species to maintain rapid growth and high nitrogen‐use efficiency even with limited phosphorus (Chen et al. 2023), conferring a significant competitive advantage in the often nitrogen‐enriched soils of urban parks (Wang et al. 2025). These results also suggest that non‐native species are lower‐nutrient content food sources for herbivores in cities. This aligns with the core prediction of the EICA hypothesis concerning resource reallocation, but challenges its classic assumption of being driven by enemy release, as no significant difference in herbivory was detected. This combination of high resource‐use efficiency, rapid growth, and reduced defensive investment represents a strategy well‐suited to the conditions of managed, often nutrient‐rich urban ecosystems.

In shrubs, our study is consistent with previous findings that non‐native species suffer less insect damage than native ones in parks (Liu et al. 2007) in supporting the prediction of ERH. Indeed, this finding is part of a broader pattern of low herbivory observed across all woody plants in our study: the average herbivory rate within urban parks in Beijing was recorded at 2.4%, which is significantly lower than the global herbivory rate observed in natural communities, typically ranging from 5% to 8% (Kozlov et al. 2015; Zhang et al. 2016, 2017). This system‐wide depression of herbivory is a stark example of urban trophic decoupling (Nelson and Forbes 2014). This phenomenon, where the linkage between producers and consumers is fundamentally weakened, reflects a breakdown in bottom‐up control (Feng et al. 2024), thereby impeding energy flow to higher trophic levels. Lower levels of energy transmission can have negative effects on the maintenance of biodiversity (e.g., butterfly and bird diversity). Given that this effect was particularly pronounced for non‐native shrubs, which are poorly integrated into the local food web, we suggest that planting more native shrubs is a key strategy to enhance food web integrity. For example, incorporating native species such as *Philadelphus pekinensis*, which attracts a wide range of pollinators, and *Lespedeza bicolor* , which supports insects and birds, could effectively bolster local biodiversity and resilience.

For trees, our findings provided an interesting test of invasion hypotheses. We did not detect a significant difference in herbivory, suggesting that a simple ERH may not apply to non‐native trees in this system. However, we observed a trait allocation pattern that is remarkably consistent with the core mechanism of the EICA hypothesis. Non‐native species tend to adopt a more rapid growth strategy (e.g., higher SLA) with higher leaf C content, lower nutrient content (e.g., lower N and P content), and lower inputs to defense (e.g., total phenols). Intriguingly, this trade‐off from defense toward growth, which resembles the pattern predicted by EICA, occurs without a detectable difference in current herbivory pressure. This suggests it may be driven by historical selective pressures or, more likely, by horticultural selection for species that grow quickly. Furthermore, these traits have direct implications for urban climate mitigation. The combination of higher leaf carbon content and a fast‐return strategy suggests these non‐native trees could be highly effective for short‐term carbon sequestration at the leaf level (Amoatey and Sulaiman 2020).

We found that the contribution of phylogenetic history and spatial variation to the variance in leaf functional traits and herbivory in non‐native species was lower, resulting in greater uncertainty in the variation of traits compared to native species. This pattern is probably due to the long‐term coevolution of native species with native phytophagous insects in the local area (Wade 2007). At the same time, our study demonstrates that leaf functional traits are predominantly influenced by phylogenetic history rather than spatial factors, highlighting the substantial homogeneity of biotic and abiotic environments within urban parks. Biotic homogenization, the process where communities become more similar (Blowes et al. 2024), is a recognized consequence of species introductions in urban ecosystems (Zeeman et al. 2017; Dylewski et al. 2023). The introduction of non‐native species frequently modifies community composition, thus promoting this trend of homogenization (Li et al. 2018). Given that non‐native species generally exhibit lower phylogenetic diversity compared to native species (Ricotta et al. 2009), they are particularly prone to driving homogenization in urban settings. Horticultural selection in cities could paradoxically lead to both trait divergence in esthetic qualities valued for novelty (Hu et al. 2023) and trait convergence in functional qualities required for survival (Bayón et al. 2021; Milanović et al. 2021). As we all know, biotic homogenization in cities is a globally widespread phenomenon, and the disappearance of local biome uniqueness can have a negative impact on biodiversity and ecosystem function (Baiser et al. 2012), especially in China (Qian et al. 2016; Wang, Svenning, et al. 2021). The observed weaker phylogenetic constraints in non‐native species are consistent with the broader pattern of biotic homogenization documented in urban ecosystems, and further research could test this hypothesis using phylogenetic or functional beta diversity metrics. Non‐native species account for 27% of greening plants in Chinese cities (Qian et al. 2016).

Our results show that herbivory is less influenced by phylogenetic history than leaf functional traits, and native plants are more susceptible to phylogenetic history and spatial factors than non‐native plants. Compared to leaf functional traits, herbivory involves much more complex interactions, including interactions between plants and phytophagous insects (Meineke et al. 2019; Barreto et al. 2021; Těšitel et al. 2021), the bottom‐up effect (Zhang et al. 2017) as well as the top‐down effect (Letourneau et al. 2009; Kozlov et al. 2017). These complex interactions could make herbivory highly variable and challenging to predict. We also acknowledge that our focus on mature leaves provides a static snapshot, as herbivory levels can vary significantly with leaf age. Therefore, it is crucial to conduct further studies to explore these dynamics across different ecological contexts and larger scales.

Our findings, which offer an ecological cross‐sectional view, also compel us to consider the ongoing eco‐evolutionary dynamics within these novel urban ecosystems. Cities are not merely new habitats but potent evolutionary arenas, where stressors like the heat‐island effect and soil compaction (Johnson and Munshi‐South 2017) can accelerate trait changes (Borowy and Swan 2020). The distinct trait syndromes we identified are likely part of these dynamics. For instance, the successful enemy release of non‐native shrubs could initiate an evolutionary trajectory toward even lower defense that further decouples them from local food webs. Similarly, the strategy of rapid growth and low defense exhibited by non‐native trees will inevitably alter soil nutrient cycling (Joswig et al. 2022) through their litter, creating new environmental filters that in turn exert new selective pressures on the plant community. Notably, the fragmented nature of urban landscapes may amplify these feedbacks, turning isolated parks into evolutionary islands where rapid local adaptation can occur on decadal rather than millennial timescales (Alberti et al. 2020). This rapid urban evolution is now a well‐documented global phenomenon (Santangelo, Ness, et al. 2022). Therefore, a valuable direction for future research would be to use common park experiments across urbanization gradients to explicitly test how urban conditions drive plant adaptation to cities.

In light of these, we suggest that the government should highlight heterogeneity and monitor key ecological functions (Chang and Lee 2016; Santangelo, Roux, and Johnson 2022). This includes using native shrubs to support local food webs and maintain biodiversity, incorporating non‐native trees for their rapid growth and carbon capture, while carefully selecting species to minimize ecological drawbacks.

## 5 Conclusion

Our study revealed that non‐native woody species in Beijing's urban parks adopt distinct functional strategies compared to native species, characterized by lower leaf nutrient contents, reduced physical defenses, and a fast‐return growth strategy. Non‐native shrubs experienced lower herbivory, supporting the ERH; but non‐native trees, despite showing EICA‐consistent traits (e.g., reduced defense, increased resource acquisition), did not, indicating only partial applicability of these hypotheses. Additionally, trait variation in non‐native species was less influenced by phylogenetic history and spatial variation, promoting biotic homogenization and risking urban biodiversity. We recommend incorporating more native shrubs to sustain local food webs. Although non‐native trees may offer short‐term carbon sequestration, their uncertain long‐term effects and potential to drive homogenization call for caution in selection. Given the complexity of herbivory and our focus on Beijing, a temperate megacity, further studies integrating long‐term monitoring with multi‐layered data across climatic zones are needed to clarify the ecological impacts of non‐native species and guide sustainable biodiversity management.

## Author Contributions

**Yingjie Wang:** conceptualization (equal), investigation (equal), methodology (equal), software (equal), visualization (equal), writing – original draft (equal), writing – review and editing (equal). **Shuang Zhang:** funding acquisition (equal), writing – review and editing (equal). **Xingwu Duan:** supervision (equal), writing – review and editing (equal). **Keming Ma:** supervision (equal), writing – review and editing (equal).

## Conflicts of Interest

The authors declare no conflicts of interest.

## Supporting information


**Tables S1–S4:** ece371947‐sup‐0001‐Tables.docx.

**Data S1:** ece371947‐sup‐0002‐DataS1.xlsx.

## Data Availability

All the required data are uploaded as Supporting Information.

## References

[ece371947-bib-0002] Alberti, M. , E. P. Palkovacs , S. D. Roches , et al. 2020. “The Complexity of Urban Eco‐Evolutionary Dynamics.” Bioscience 70: 772–793.

[ece371947-bib-0003] Amoatey, P. , and H. Sulaiman . 2020. “Quantifying Carbon Storage Potential of Urban Plantations and Landscapes in Muscat, Oman.” Environment, Development and Sustainability 22: 7969–7984.

[ece371947-bib-0005] Baiser, B. , J. D. Olden , S. Record , J. L. Lockwood , and M. L. McKinney . 2012. “Pattern and Process of Biotic Homogenization in the New Pangaea.” Proceedings of the Royal Society B: Biological Sciences 279: 4772–4777. 10.1098/rspb.2012.1651 PMC3497087 23055062

[ece371947-bib-0006] Bardgett, R. D. 2017. “Plant Trait‐Based Approaches for Interrogating Belowground Function.” Biology and Environment: Proceedings of the Royal Irish Academy 117B: 1–13.

[ece371947-bib-0007] Barreto, J. R. , E. Berenguer , J. Ferreira , et al. 2021. “Assessing Invertebrate Herbivory in Human‐Modified Tropical Forest Canopies.” Ecology and Evolution 11: 4012–4022. 33976790 10.1002/ece3.7295 PMC8093672

[ece371947-bib-0008] Bates, D. , M. Mächler , B. Bolker , and S. Walker . 2015. “Fitting Linear Mixed‐Effects Models Using lme4.” Journal of Statistical Software 67: 1–48.

[ece371947-bib-0009] Bayón, Á. , O. Godoy , N. Maurel , M. van Kleunen , and M. Vilà . 2021. “Proportion of Non‐Native Plants in Urban Parks Correlates With Climate, Socioeconomic Factors and Plant Traits.” Urban Forestry & Urban Greening 63: 127215.

[ece371947-bib-0011] Blossey, B. , and R. Notzold . 1995. “Evolution of Increased Competitive Ability in Invasive Nonindigenous Plants: A Hypothesis.” Journal of Ecology 83: 887–889.

[ece371947-bib-0012] Blowes, S. A. , B. McGill , V. Brambilla , et al. 2024. “Synthesis Reveals Approximately Balanced Biotic Differentiation and Homogenization.” Science Advances 10: eadj9395. 38381832 10.1126/sciadv.adj9395 PMC10881054

[ece371947-bib-0013] Borowy, D. , and C. M. Swan . 2020. “A Multi‐Trait Comparison of an Urban Plant Species Pool Reveals the Importance of Intraspecific Trait Variation and Its Influence on Distinct Functional Responses to Soil Quality.” Frontiers in Ecology and Evolution 8: 68.

[ece371947-bib-0014] Bossdorf, O. 2013. “Enemy Release and Evolution of Increased Competitive Ability: At Last, a Smoking Gun!” New Phytologist 198: 638–640. 23577595 10.1111/nph.12265

[ece371947-bib-0015] Burns, J. H. 2006. “Relatedness and Environment Affect Traits Associated With Invasive and Noninvasive Introduced Commelinaceae.” Ecological Applications 16: 1367–1376. 16937804 10.1890/1051-0761(2006)016[1367:raeata]2.0.co;2

[ece371947-bib-0016] Callaway, R. M. , J. E. Lucero , J. L. Hierro , and C. J. Lortie . 2022. “The EICA Is Dead? Long Live the EICA!” Ecology Letters 25: 2289–2302. 35986512 10.1111/ele.14088

[ece371947-bib-0017] Callaway, R. M. , and W. M. Ridenour . 2004. “Novel Weapons: Invasive Success and the Evolution of Increased Competitive Ability.” Frontiers in Ecology and the Environment 2: 436–443.

[ece371947-bib-0018] Cary, K. L. , and J. Pittermann . 2018. “Small Trees, Big Problems: Comparative Leaf Function Under Extreme Edaphic Stress.” American Journal of Botany 105: 50–59. 29532934 10.1002/ajb2.1007

[ece371947-bib-0019] Castro‐Diez, P. , O. Godoy , A. Alonso , A. Gallardo , and A. Saldana . 2014. “What Explains Variation in the Impacts of Exotic Plant Invasions on the Nitrogen Cycle? A Meta‐Analysis.” Ecology Letters 17: 1–12. 24134461 10.1111/ele.12197

[ece371947-bib-0020] Chang, H.‐Y. , and Y.‐F. Lee . 2016. “Effects of Area Size, Heterogeneity, Isolation, and Disturbances on Urban Park Avifauna in a Highly Populated Tropical City.” Urban Ecosystems 19: 257–274.

[ece371947-bib-0021] Chen, S. , W. Zhang , X. Ge , et al. 2023. “Response of Plant and Soil N, P, and N:P Stoichiometry to N Addition in China: A Meta‐Analysis.” Plants (Basel) 12: 2104. 37299084 10.3390/plants12112104 PMC10255806

[ece371947-bib-0022] Colautti, R. I. , A. Ricciardi , I. A. Grigorovich , and H. J. MacIsaac . 2004. “Is Invasion Success Explained by the Enemy Release Hypothesis?” Ecology Letters 7: 721–733.

[ece371947-bib-0024] Craine, J. M. , and W. G. Lee . 2003. “Covariation in Leaf and Root Traits for Native and Non‐Native Grasses Along an Altitudinal Gradient in New Zealand.” Oecologia 134: 471–478. 12647118 10.1007/s00442-002-1155-6

[ece371947-bib-0026] de Souza e Silva, J. L. , M. T. P. de Oliveira , W. Oliveira , L. A. Borges , O. Cruz‐Neto , and A. V. Lopes . 2020. “High Richness of Exotic Trees in Tropical Urban Green Spaces: Reproductive Systems, Fruiting and Associated Risks to Native Species.” Urban Forestry & Urban Greening 50: 126659.

[ece371947-bib-0027] Díaz de León Guerrero, S. D. , G. González‐Rebeles Guerrero , T. M. Ibarra‐Montes , et al. 2020. “Functional Traits Indicate Faster Resource Acquisition for Alien Herbs Than Native Shrubs in an Urban Mediterranean Shrubland.” Biological Invasions 22: 2699–2712.

[ece371947-bib-0028] Durand, L. Z. , and G. Goldstein . 2001. “Growth, Leaf Characteristics, and Spore Production in Native and Invasive Tree Ferns in Hawaii.” American Fern Journal 91: 25–35.

[ece371947-bib-0029] Dylewski, Ł. , W. Banaszak‐Cibicka , Ł. Maćkowiak , and M. K. Dyderski . 2023. “How Do Urbanization and Alien Species Affect the Plant Taxonomic, Functional, and Phylogenetic Diversity in Different Types of Urban Green Areas?” Environmental Science and Pollution Research 30: 92390–92403. 37491488 10.1007/s11356-023-28808-y PMC10447280

[ece371947-bib-0030] Feng, Z. , R. Marsland III , J. W. Rocks , and P. Mehta . 2024. “Emergent Competition Shapes Top‐Down Versus Bottom‐Up Control in Multi‐Trophic Ecosystems.” PLoS Computational Biology 20: e1011675. 38330086 10.1371/journal.pcbi.1011675 PMC10852287

[ece371947-bib-0031] Fickenscher, J. L. , J. A. Litvaitis , T. D. Lee , and P. C. Johnson . 2014. “Insect Responses to Invasive Shrubs: Implications to Managing Thicket Habitats in the Northeastern United States.” Forest Ecology and Management 322: 127–135.

[ece371947-bib-0032] Frank, S. D. 2014. “Bad Neighbors: Urban Habitats Increase Cankerworm Damage to Non‐Host Understory Plants.” Urban Ecosystems 17: 1135–1145.

[ece371947-bib-0033] Grunzweig, L. , D. J. Spiering , A. Labatore , and R. J. Warren . 2015. “Non‐Native Plant Invader Renders Suitable Habitat Unsuitable.” Arthropod‐Plant Interactions 9: 577–583.

[ece371947-bib-0034] Gulias, J. , J. Flexas , M. Mus , J. Cifre , E. Lefi , and H. Medrano . 2003. “Relationship Between Maximum Leaf Photosynthesis, Nitrogen Content and Specific Leaf Area in Balearic Endemic and Non‐Endemic Mediterranean Species.” Annals of Botany 92: 215–222. 12805082 10.1093/aob/mcg123 PMC4243646

[ece371947-bib-0036] Halliwell, B. , B. R. Holland , and L. A. Yates . 2025. “Multi‐Response Phylogenetic Mixed Models: Concepts and Application.” Biological Reviews 100: 1294–1316. 40192008 10.1111/brv.70001 PMC12120399

[ece371947-bib-0038] Heberling, J. M. , and J. D. Fridley . 2013. “Resource‐Use Strategies of Native and Invasive Plants in Eastern North American Forests.” New Phytologist 200: 523–533. 23815090 10.1111/nph.12388

[ece371947-bib-0039] Hernández‐Montes, E. , M. Tomás , J. M. Escalona , J. Bota , and H. Medrano . 2019. “Leaf Growth Rate and Nitrogen Content Determine Respiratory Costs During Leaf Expansion in Grapevines.” Physiologia Plantarum 165: 746–754. 29885063 10.1111/ppl.12769

[ece371947-bib-0040] Hu, S. , C. Jin , R. Liao , et al. 2023. “Herbaceous Ornamental Plants With Conspicuous Aesthetic Traits Contribute to Plant Invasion Risk in Subtropical Urban Parks.” Journal of Environmental Management 347: 119059. 37769469 10.1016/j.jenvman.2023.119059

[ece371947-bib-0041] Hu, Y. J. , X. B. Kong , J. Zheng , J. Sun , L. L. Wang , and M. Z. Min . 2018. “Urban Expansion and Farmland Loss in Beijing During 1980–2015.” Sustainability 10: 3927.

[ece371947-bib-0042] Hulme, P. E. , and M. Bernard‐Verdier . 2018. “Comparing Traits of Native and Alien Plants: Can We Do Better?” Functional Ecology 32: 117–125.

[ece371947-bib-0043] Jin, Y. , and H. Qian . 2019. “V.PhyloMaker: An R Package That Can Generate Very Large Phylogenies for Vascular Plants.” Ecography 42: 1353–1359. 10.1016/j.pld.2022.05.005 PMC9363651 35967255

[ece371947-bib-0044] Johnson, M. T. J. , and J. Munshi‐South . 2017. “Evolution of Life in Urban Environments.” Science 358: eaam8327. 29097520 10.1126/science.aam8327

[ece371947-bib-0045] Joswig, J. S. , C. Wirth , M. C. Schuman , et al. 2022. “Climatic and Soil Factors Explain the Two‐Dimensional Spectrum of Global Plant Trait Variation.” Nature Ecology & Evolution 6: 36–50. 34949824 10.1038/s41559-021-01616-8 PMC8752441

[ece371947-bib-0046] Keane, R. M. , and M. J. Crawley . 2002. “Exotic Plant Invasions and the Enemy Release Hypothesis.” Trends in Ecology & Evolution 17: 164–170.

[ece371947-bib-0047] Khan, N. , M. K. Jhariya , D. K. Yadav , and A. Banerjee . 2020. “Structure, Diversity and Ecological Function of Shrub Species in an Urban Setup of Sarguja, Chhattisgarh, India.” Environmental Science and Pollution Research 27: 5418–5432. 31848969 10.1007/s11356-019-07172-w

[ece371947-bib-0048] Kobe, R. K. , C. A. Lepczyk , and M. Iyer . 2005. “Resorption Efficiency Decreases With Increasing Green Leaf Nutrients in a Global Data Set.” Ecology 86: 2780–2792.

[ece371947-bib-0049] Kozlov, M. V. , V. Lanta , V. Zverev , K. Rainio , M. A. Kunavin , and E. L. Zvereva . 2017. “Decreased Losses of Woody Plant Foliage to Insects in Large Urban Areas Are Explained by Bird Predation.” Global Change Biology 23: 4354–4364. 28317226 10.1111/gcb.13692

[ece371947-bib-0050] Kozlov, M. V. , V. Lanta , V. Zverev , and E. L. Zvereva . 2015. “Global Patterns in Background Losses of Woody Plant Foliage to Insects.” Global Ecology and Biogeography 24: 1126–1135.

[ece371947-bib-0051] Kozlov, M. V. , and E. L. Zvereva . 2017. “Background Insect Herbivory: Impacts, Patterns and Methodology.” In Progress in Botany, edited by U. L. Francisco , M. Cánovas , and R. Matyssek , 313–355. Springer International Publishing.

[ece371947-bib-0052] Kubelka, V. , M. Šálek , P. Tomkovich , Z. Végvári , R. P. Freckleton , and T. Székely . 2018. “Global Pattern of Nest Predation Is Disrupted by Climate Change in Shorebirds.” Science 362: 680–683. 30409881 10.1126/science.aat8695

[ece371947-bib-0053] Lai, H. , E. J. Flies , P. Weinstein , and A. Woodward . 2019. “The Impact of Green Space and Biodiversity on Health.” Frontiers in Ecology and the Environment 17: 383–390.

[ece371947-bib-0054] Lake, J. C. , and M. R. Leishman . 2004. “Invasion Success of Exotic in Natural Ecosystems: The Role of Disturbance, Plant Attributes and Freedom From Herbivores.” Biological Conservation 117: 215–226.

[ece371947-bib-0055] Leffler, A. J. , J. J. James , T. A. Monaco , and R. L. Sheley . 2014. “A New Perspective on Trait Differences Between Native and Invasive Exotic Plants.” Ecology 95: 298–305. 24669724 10.1890/13-0102.1

[ece371947-bib-0056] Leishman, M. R. , T. Haslehurst , A. Ares , and Z. Baruch . 2007. “Leaf Trait Relationships of Native and Invasive Plants: Community‐ and Global‐Scale Comparisons.” New Phytologist 176: 635–643. 17822409 10.1111/j.1469-8137.2007.02189.x

[ece371947-bib-0057] Lenth, R. V. , B. Banfai , B. Bolker , et al. 2025. “Emmeans: Estimated Marginal Means, Aka Least‐Squares Means.” R Package Version 1.10.2.

[ece371947-bib-0058] Letourneau, D. K. , J. A. Jedlicka , S. G. Bothwell , and C. R. Moreno . 2009. “Effects of Natural Enemy Biodiversity on the Suppression of Arthropod Herbivores in Terrestrial Ecosystems.” Annual Review of Ecology, Evolution, and Systematics 40: 573–592.

[ece371947-bib-0059] Li, D. , J. L. Lockwood , and B. Baiser . 2018. “Taxonomic and Phylogenetic Homogenization Across Us National Parks: The Role of Non‐Native Species.” In From Biocultural Homogenization to Biocultural Conservation, edited by R. Rozzi , R. H. May Jr. , F. S. Chapin III , et al., 275–288. Springer International Publishing.

[ece371947-bib-0060] Liñán‐Vigo, F. , and J. Núñez‐Farfán . 2024. “Plasticity in Biomass Allocation Underlies Tolerance to Leaf Damage in Native and Non‐Native Populations of *Datura stramonium* .” Oecologia 205: 613–626. 39048862 10.1007/s00442-024-05585-0 PMC11358249

[ece371947-bib-0061] Liu, H. , and P. Stiling . 2006. “Testing the Enemy Release Hypothesis: A Review and Meta‐Analysis.” Biological Invasions 8: 1535–1545.

[ece371947-bib-0062] Liu, H. , P. Stiling , and R. W. Pemberton . 2007. “Does Enemy Release Matter for Invasive Plants? Evidence From a Comparison of Insect Herbivore Damage Among Invasive, Non‐Invasive and Native Congeners.” Biological Invasions 9: 773–781.

[ece371947-bib-0063] Liu, M.‐C. , D.‐L. Kong , X.‐R. Lu , et al. 2017. “Higher Photosynthesis, Nutrient‐ and Energy‐Use Efficiencies Contribute to Invasiveness of Exotic Plants in a Nutrient Poor Habitat in Northeast China.” Physiologia Plantarum 160: 373–382. 28321883 10.1111/ppl.12566

[ece371947-bib-0064] Mata, L. , A. N. Andersen , A. Morán‐Ordóñez , et al. 2021. “Indigenous Plants Promote Insect Biodiversity in Urban Greenspaces.” Ecological Applications 31: e02309. 33605502 10.1002/eap.2309

[ece371947-bib-0065] Mata, L. , C. E. Ramalho , J. Kennedy , et al. 2020. “Bringing Nature Back Into Cities.” People and Nature 2: 350–368.

[ece371947-bib-0066] Matter, S. F. , J. R. Brzyski , C. J. Harrison , et al. 2012. “Invading From the Garden? A Comparison of Leaf Herbivory for Exotic and Native Plants in Natural and Ornamental Settings.” Insect Science 19: 677–682.

[ece371947-bib-0067] Mediavilla, S. , J. Babiano , M. Martínez‐Ortega , and A. Escudero . 2018. “Ontogenetic Changes in Anti‐Herbivore Defensive Traits in Leaves of Four Mediterranean Co‐Occurring *Quercus* Species.” Ecological Research 33: 1093–1102.

[ece371947-bib-0068] Meineke, E. K. , A. T. Classen , N. J. Sanders , and T. Jonathan Davies . 2019. “Herbarium Specimens Reveal Increasing Herbivory Over the Past Century.” Journal of Ecology 107: 105–117.

[ece371947-bib-0069] Milanović, M. , I. Kühn , P. Pyšek , and S. Knapp . 2021. “Functional Diversity Changes in Native and Alien Urban Flora Over Three Centuries.” Biological Invasions 23: 2337–2353.

[ece371947-bib-0070] Montesinos, D. 2022. “Fast Invasives Fastly Become Faster: Invasive Plants Align Largely With the Fast Side of the Plant Economics Spectrum.” Journal of Ecology 110: 1010–1014.

[ece371947-bib-0071] National Bureau of Statistics of China . 2022. China Statistical Yearbook 2022. China Statistics Press.

[ece371947-bib-0072] Nelson, A. E. , and A. A. Forbes . 2014. “Urban Land Use Decouples Plant‐Herbivore‐Parasitoid Interactions at Multiple Spatial Scales.” PLoS One 9: e102127. 25019962 10.1371/journal.pone.0102127 PMC4096920

[ece371947-bib-0073] Occhipinti, A. 2013. “Plant Coevolution: Evidences and New Challenges.” Journal of Plant Interactions 8: 188–196.

[ece371947-bib-0074] Ordonez, A. 2014. “Functional and Phylogenetic Similarity of Alien Plants to Co‐Occurring Natives.” Ecology 95: 1191–1202. 25000751 10.1890/13-1002.1

[ece371947-bib-0075] Orme, D. , R. Freckleton , G. Thomas , et al. 2023. “caper: Comparative Analyses of Phylogenetics and Evolution in R.”

[ece371947-bib-0076] Padullés Cubino, J. , D. Borowy , S. Knapp , et al. 2021. “Contrasting Impacts of Cultivated Exotics on the Functional Diversity of Domestic Gardens in Three Regions With Different Aridity.” Ecosystems 24: 875–890.

[ece371947-bib-0077] Parsons, S. E. , L. M. Kerner , and S. D. Frank . 2020. “Effects of Native and Exotic Congeners on Diversity of Invertebrate Natural Enemies, Available Spider Biomass, and Pest Control Services in Residential Landscapes.” Biodiversity and Conservation 29: 1241–1262.

[ece371947-bib-0078] Pattison, R. R. , G. Goldstein , and A. Ares . 1998. “Growth, Biomass Allocation and Photosynthesis of Invasive and Native Hawaiian Rainforest Species.” Oecologia 117: 449–459. 28307669 10.1007/s004420050680

[ece371947-bib-0079] Peeters, P. J. 2002. “Correlations Between Leaf Structural Traits and the Densities of Herbivorous Insect Guilds.” Biological Journal of the Linnean Society 77: 43–65.

[ece371947-bib-0080] Perez‐Harguindeguy, N. , S. Diaz , E. Garnier , et al. 2013. “New Handbook for Standardised Measurement of Plant Functional Traits Worldwide.” Australian Journal of Botany 61: 167–234.

[ece371947-bib-0081] Prior, K. M. , T. H. Q. Powell , A. L. Joseph , and J. J. Hellmann . 2015. “Insights From Community Ecology Into the Role of Enemy Release in Causing Invasion Success: The Importance of Native Enemy Effects.” Biological Invasions 17: 1283–1297.

[ece371947-bib-0082] Pyšek, P. , A. M. Manceur , C. Alba , et al. 2015. “Naturalization of Central European Plants in North America: Species Traits, Habitats, Propagule Pressure, Residence Time.” Ecology 96: 762–774. 26236872 10.1890/14-1005.1

[ece371947-bib-0083] Qian, S. , M. Qi , L. Huang , L. Zhao , D. Lin , and Y. Yang . 2016. “Biotic Homogenization of China's Urban Greening: A Meta‐Analysis on Woody Species.” Urban Forestry & Urban Greening 18: 25–33.

[ece371947-bib-0084] Revell, L. J. 2012. “Phytools: An R Package for Phylogenetic Comparative Biology (And Other Things).” Methods in Ecology and Evolution 3: 217–223.

[ece371947-bib-0085] Ricotta, C. , F. A. La Sorte , P. Pyšek , G. L. Rapson , L. Celesti‐Grapow , and K. Thompson . 2009. “Phyloecology of Urban Alien Floras.” Journal of Ecology 97: 1243–1251.

[ece371947-bib-0086] Sandel, B. , and R. Low . 2019. “Intraspecific Trait Variation, Functional Turnover and Trait Differences Among Native and Exotic Grasses Along a Precipitation Gradient.” Journal of Vegetation Science 30: 633–643.

[ece371947-bib-0087] Santangelo, J. S. , R. W. Ness , B. Cohan , et al. 2022. “Global Urban Environmental Change Drives Adaptation in White Clover.” Science 375: 1275–1281. 35298255 10.1126/science.abk0989

[ece371947-bib-0088] Santangelo, J. S. , C. Roux , and M. T. J. Johnson . 2022. “The Effects of Environmental Heterogeneity Within a City on the Evolution of Clines.” Journal of Ecology 110: 2950–2959.

[ece371947-bib-0089] Santos, O. d. A. , S. R. Marques Couceiro , A. C. Cavalcante Rezende , and M. D. de Sousa Silva . 2016. “Composition and Richness of Woody Species in Riparian Forests in Urban Areas of Manaus, Amazonas, Brazil.” Landscape and Urban Planning 150: 70–78.

[ece371947-bib-0090] Schwarz, N. , M. Moretti , M. N. Bugalho , et al. 2017. “Understanding Biodiversity‐Ecosystem Service Relationships in Urban Areas: A Comprehensive Literature Review.” Ecosystem Services 27: 161–171.

[ece371947-bib-0091] Seastedt, T. R. , and P. Pysek . 2011. “Mechanisms of Plant Invasions of North America and European Grasslands.” In Annual Review of Ecology, Evolution, and Systematics, edited by D. J. Futuyma , H. B. Shaffer , and D. Simberloff , vol. 42, 133–153. Annual Reviews.

[ece371947-bib-0092] Smith, M. D. , and A. K. Knapp . 2001. “Physiological and Morphological Traits of Exotic, Invasive Exotic, and Native Plant Species in Tallgrass Prairie.” International Journal of Plant Sciences 162: 785–792.

[ece371947-bib-0093] Tallamy, D. W. , M. Ballard , and V. D'Amico . 2010. “Can Alien Plants Support Generalist Insect Herbivores?” Biological Invasions 12: 2285–2292.

[ece371947-bib-0094] Tartaglia, E. S. , and M. F. J. Aronson . 2024. “Plant Native: Comparing Biodiversity Benefits, Ecosystem Services Provisioning, and Plant Performance of Native and Non‐Native Plants in Urban Horticulture.” Urban Ecosystems 27: 2587–2611.

[ece371947-bib-0095] Těšitel, J. , M. Tahadlová , J. Lepš , and N. Hölzel . 2021. “Linking Insect Herbivory With Plant Traits: Phylogenetically Structured Trait Syndromes Matter.” Journal of Vegetation Science 32: e13061.

[ece371947-bib-0096] Threlfall, C. G. , A. Ossola , A. K. Hahs , N. S. G. Williams , L. Wilson , and S. J. Livesley . 2016. “Variation in Vegetation Structure and Composition Across Urban Green Space Types.” Frontiers in Ecology and Evolution 4: 66.

[ece371947-bib-0097] Wade, M. J. 2007. “The Co‐Evolutionary Genetics of Ecological Communities.” Nature Reviews Genetics 8: 185–195. 10.1038/nrg2031 17279094

[ece371947-bib-0098] Wang, J. , Q. Yang , T. Zhou , Z. Wang , and B. Yu . 2025. “Ecological Stoichiometry Characteristics and Influencing Factors of Soil Carbon, Nitrogen, and Phosphorus in Green Spaces Along the Urban‐To‐Rural Gradient of Nanchang, China.” Forests 16: 644.

[ece371947-bib-0099] Wang, S. , Z. Xiao , T. Yang , M. Jiang , and X. Wei . 2021. “Shifts in Leaf Herbivory Stress and Defense Strategies of Endangered Tree Species After 20–35 Years of Ex‐Situ Conservation.” Global Ecology and Conservation 26: e01490.

[ece371947-bib-0100] Wang, X. , J.‐C. Svenning , J. Liu , et al. 2021. “Regional Effects of Plant Diversity and Biotic Homogenization in Urban Greenspace—The Case of University Campuses Across China.” Urban Forestry & Urban Greening 62: 127170.

[ece371947-bib-0101] War, A. R. , G. K. Taggar , B. Hussain , M. S. Taggar , R. M. Nair , and H. C. Sharma . 2018. “Plant Defence Against Herbivory and Insect Adaptations.” AoB Plants 10: ply037.

[ece371947-bib-0102] Warton, D. I. , and F. K. C. Hui . 2011. “The Arcsine Is Asinine: The Analysis of Proportions in Ecology.” Ecology 92: 3–10. 21560670 10.1890/10-0340.1

[ece371947-bib-0103] Waterman, P. G. , and S. Mole . 1994. Analysis of Phenolic Plant Metabolites. Blackwell Science Publications.

[ece371947-bib-0104] White, E. M. , N. M. Sims , and A. R. Clarke . 2008. “Test of the Enemy Release Hypothesis: The Native Magpie Moth Prefers a Native Fireweed (*Senecio pinnatifolius*) to Its Introduced Congener ( *S. madagascariensis* ).” Austral Ecology 33: 110–116.

[ece371947-bib-0105] Wright, I. J. , P. B. Reich , M. Westoby , et al. 2004. “The Worldwide Leaf Economics Spectrum.” Nature 428: 821–827. 15103368 10.1038/nature02403

[ece371947-bib-0106] Xiao, C. , B. Liu , C. Wu , et al. 2022. “A Dataset on Inventory and Geographical Distributions of Vascular Plants in Beijing, China.” Biodiversity Science 30: 5–13.

[ece371947-bib-0107] Xiao, Y. , S. Liu , M. Zhang , et al. 2021. “Plant Functional Groups Dominate Responses of Plant Adaptive Strategies to Urbanization.” Frontiers in Plant Science 12: 773676. 34917107 10.3389/fpls.2021.773676 PMC8669269

[ece371947-bib-0108] Xirocostas, Z. A. , S. A. Debono , E. Slavich , and A. T. Moles . 2022. “The ZAX Herbivory Trainer‐Free Software for Training Researchers to Visually Estimate Leaf Damage.” Methods in Ecology and Evolution 13: 596–602.

[ece371947-bib-0109] Xu, L. , N. Zhang , T. Wei , et al. 2023. “Adaptation Strategies of Leaf Traits and Leaf Economic Spectrum of Two Urban Garden Plants in China.” BMC Plant Biology 23: 274. 37221486 10.1186/s12870-023-04301-z PMC10207826

[ece371947-bib-0110] Yu, L. R. , Z. C. Zhu , and X. Y. Pan . 2020. “Phenotypic Plasticity of *Alternanthera philoxeroides* in Response to Root Neighbors of Kin: Introduced vs. Native Genotypes.” Biodiversity Science 28: 651–657.

[ece371947-bib-0111] Zeeman, B. J. , M. J. McDonnell , D. Kendal , and J. W. Morgan . 2017. “Biotic Homogenization in an Increasingly Urbanized Temperate Grassland Ecosystem.” Journal of Vegetation Science 28: 550–561.

[ece371947-bib-0112] Zeeman, B. J. , V. Minden , and J. W. Morgan . 2018. “Non‐Native Plant Cover and Functional Trait Composition of Urban Temperate Grasslands in Relation to Local‐ and Landscape‐Scale Road Density.” Biological Invasions 20: 3025–3036.

[ece371947-bib-0113] Zhang, S. , G.‐R. Xu , Y.‐X. Zhang , W.‐F. Zhang , and M. Cao . 2023. “Canopy Height, Rather Than Neighborhood Effects, Shapes Leaf Herbivory in a Tropical Rainforest.” Ecology 104: e4028. 36898962 10.1002/ecy.4028

[ece371947-bib-0114] Zhang, S. , Y. Zhang , and K. Ma . 2016. “Latitudinal Variation in Herbivory: Hemispheric Asymmetries and the Role of Climatic Drivers.” Journal of Ecology 104: 1089–1095.

[ece371947-bib-0115] Zhang, S. , Y. Zhang , and K. Ma . 2017. “The Association of Leaf Lifespan and Background Insect Herbivory at the Interspecific Level.” Ecology 98: 425–432. 27861782 10.1002/ecy.1649

[ece371947-bib-0116] Ziyatdinov, A. , M. Vazquez‐Santiago , H. Brunel , A. Martinez‐Perez , H. Aschard , and J. M. Soria . 2018. “Ime4qtl: Linear Mixed Models With Flexible Covariance Structure for Genetic Studies of Related Individuals.” BMC Bioinformatics 19: 68. 29486711 10.1186/s12859-018-2057-x PMC5830078

