# Correction to “Non‐Native Woody Plant Species Show Different Leaf Functional Traits and Herbivory Levels From Native Ones in the Urban Areas of Beijing, China”

**DOI:** 10.1002/ece3.72130

**Published:** 2025-09-23

Wang, Y., S. Zhang, X. Duan, and K. Ma. 2025. “Non‐Native Woody Plant Species Show Different Leaf Functional Traits and Herbivory Levels From Native Ones in the Urban Areas of Beijing, China.” *Ecology and Evolution* 15, no. 8: e71947. https://doi.org/10.1002/ece3.71947.

In the Abstract, the sentence:

“The nutrient contents, defensive traits, and levels of herbivory were measured in 2681 **leaves** across 138 (52 native and 86 non‐native species) woody plant species.”

was incorrect.

This should have read:

“The nutrient contents, defensive traits, and levels of herbivory were measured in 2681 **plants** across 138 (52 native and 86 non‐native species) woody plant species.”

The dataset consisted of over 20,000 leaves, averaged at the plant level, resulting in 2681 plants as the unit of analysis.

In the Results section, the sentence:

“In total, we collected 26,165 leaves from **2682** individual plants belonging to 36 families, 83 genera, and 138 species (61 shrub species and 77 tree species).” was incorrect.

This should have read:

“In total, we collected 26,165 leaves from **2681** individual plants belonging to 36 families, 83 genera, and 138 species (61 shrub species and 77 tree species).”

These corrections do not affect the results, interpretation, or conclusions of the study.

The online version of this article has been corrected accordingly.

We apologize for this error.

