# Supplementary material for: Non‐Native Woody Plant Species Show Different Leaf Functional Traits and Herbivory Levels From Native Ones in the Urban Areas of Beijing, China
Source: Ecol Evol. 2025 Aug 8;15(8):e71947. doi: 10.1002/ece3.71947 (PMC12334854; doi:10.1002/ece3.71947)
Supplement: Supplementary file 1 — Tables S1–S4: ece371947‐sup‐0001‐Tables.docx. [file ECE3-15-e71947-s001.docx]

Supplementary Material

Supplementary Table S1. Pairwise contrasts between native and non-native species for each trait within shrub and tree life forms.

| **Trait** | **Life form** | **Comparison** | **Estimate** | **SE** | **t.ratio** | ***p*.value** |
| --- | --- | --- | --- | --- | --- | --- |
| C | shrub | native –  non-native | 0.0205 | 0.00335 | 6.129 | **<.0001** |
|  | tree |  | -0.0139 | 0.00272 | -5.095 | **<.0001** |
| N | shrub | native –  non-native | -0.0226 | 0.0142 | -1.598 | 0.1101 |
|  | tree |  | 0.0244 | 0.0116 | 2.107 | **0.0352** |
| P | shrub | native –  non-native | 0.021 | 0.0245 | 0.856 | 0.3921 |
|  | tree |  | 0.0959 | 0.0201 | 4.781 | **<.0001** |
| C/N | shrub | native –  non-native | 0.0429 | 0.0149 | 2.882 | **0.004** |
|  | tree |  | -0.0379 | 0.0121 | -3.117 | **0.0018** |
| N/P | shrub | native –  non-native | -0.0436 | 0.0238 | -1.834 | 0.0668 |
|  | tree |  | -0.0692 | 0.0194 | -3.568 | **0.0004** |
| LA | shrub | native –  non-native | 0.12234 | 0.0626 | 1.953 | 0.0509 |
|  | tree |  | 0.00349 | 0.0508 | 0.069 | 0.9451 |
| SLA | shrub | native –  non-native | -0.0067 | 0.0224 | -0.299 | 0.7651 |
|  | tree |  | -0.0559 | 0.0183 | -3.055 | **0.0023** |
| Totalphenol | shrub | native –  non-native | -0.149 | 0.0876 | -1.699 | 0.0897 |
|  | tree |  | 0.245 | 0.0721 | 3.398 | **0.0007** |
| Herbivory | shrub | native –  non-native | 0.1909 | 0.0791 | 2.414 | **0.0159** |
|  | tree |  | -0.0828 | 0.0651 | -1.272 | 0.2035 |

Note: Trait values were log-transformed (log) or logit-transformed (logit) before analysis. Estimates are mean differences (native–non-native) within each life form. Significant results (p < 0.05) are in bold.

Supplementary Table S2. Main effect contrasts between shrubs and trees for each functional trait.

| **Trait** | **Comparison** | **Estimate** | **SE** | **t.ratio** | ***p*.value** |
| --- | --- | --- | --- | --- | --- |
| C | shrub - tree | 0.0125 | 0.00217 | 5.746 | **<.0001** |
| N | shrub - tree | -0.0952 | 0.00922 | -10.33 | **<.0001** |
| P | shrub - tree | 0.0976 | 0.0159 | 6.122 | **<.0001** |
| C/N | shrub - tree | 0.107 | 0.00968 | 11.089 | **<.0001** |
| N/P | shrub - tree | -0.194 | 0.0155 | -12.546 | **<.0001** |
| LA | shrub - tree | -0.882 | 0.0405 | -21.803 | **<.0001** |
| SLA | shrub - tree | -0.0842 | 0.0146 | -5.786 | **<.0001** |
| Totalphenol | shrub - tree | 0.25 | 0.057 | 4.393 | **<.0001** |
| Herbivory | shrub - tree | -0.22 | 0.0516 | -4.267 | **<.0001** |

Note: Trait values were log-transformed (log) or logit-transformed (logit) before analysis. Estimates are mean differences (shrub–tree) for each trait. Significant results (p < 0.05) are in bold.

Supplementary Table S3. Main effect contrasts between native and non-native species for each functional trait

| **Trait** | **Comparison** | **Estimate** | **SE** | **t.ratio** | ***p*.value** |
| --- | --- | --- | --- | --- | --- |
| C | native - non-native | 0.00335 | 0.00216 | 1.55 | 0.1212 |
| N | native - non-native | 0.000862 | 0.00915 | 0.094 | 0.9249 |
| P | native - non-native | 0.0584 | 0.0158 | 3.689 | **0.0002** |
| C/N | native - non-native | 0.00251 | 0.00961 | 0.261 | 0.794 |
| N/P | native - non-native | -0.0564 | 0.0153 | -3.676 | **0.0002** |
| LA | native - non-native | 0.0629 | 0.0403 | 1.56 | 0.1188 |
| SLA | native - non-native | -0.0313 | 0.0145 | -2.162 | **0.0307** |
| Totalphenol | native - non-native | 0.048 | 0.0567 | 0.848 | 0.3968 |
| Herbivory | native - non-native | 0.054 | 0.0512 | 1.055 | 0.2916 |

Note: Trait values were log-transformed (log) or logit-transformed (logit) before analysis. Estimates are mean differences (native–non-native) for each trait. Significant results (p < 0.05) are in bold.

Supplementary Table S4. Estimated marginal means (emmeans) for each functional trait by life form and species type.

| **Trait** | **Life Form** | **Comparison** | **emmean** | **SE** | **lower.CL** | **upper.CL** |
| --- | --- | --- | --- | --- | --- | --- |
| C | Shrub | native | 6.112 | 0.00338 | 6.105 | 6.119 |
|  | Shrub | non-native | 6.092 | 0.00259 | 6.086 | 6.097 |
|  | Tree | native | 6.082 | 0.00264 | 6.077 | 6.088 |
|  | Tree | non-native | 6.096 | 0.00256 | 6.091 | 6.101 |
| N | Shrub | native | 3.14 | 0.0227 | 3.1 | 3.19 |
|  | Shrub | non-native | 3.17 | 0.0207 | 3.12 | 3.21 |
|  | Tree | native | 3.26 | 0.0208 | 3.22 | 3.3 |
|  | Tree | non-native | 3.24 | 0.0206 | 3.2 | 3.28 |
| P | Shrub | native | 0.849 | 0.0303 | 0.789 | 0.909 |
|  | Shrub | non-native | 0.828 | 0.0258 | 0.777 | 0.88 |
|  | Tree | native | 0.789 | 0.026 | 0.737 | 0.841 |
|  | Tree | non-native | 0.693 | 0.0256 | 0.642 | 0.744 |
| C/N | Shrub | native | 2.97 | 0.023 | 2.92 | 3.01 |
|  | Shrub | non-native | 2.92 | 0.0208 | 2.88 | 2.97 |
|  | Tree | native | 2.82 | 0.0209 | 2.78 | 2.86 |
|  | Tree | non-native | 2.86 | 0.0207 | 2.82 | 2.9 |
| N/P | Shrub | native | 2.29 | 0.0372 | 2.22 | 2.37 |
|  | Shrub | non-native | 2.34 | 0.0338 | 2.27 | 2.4 |
|  | Tree | native | 2.47 | 0.034 | 2.41 | 2.54 |
|  | Tree | non-native | 2.54 | 0.0337 | 2.47 | 2.61 |
| LA | Shrub | native | 2.36 | 0.0587 | 2.24 | 2.47 |
|  | Shrub | non-native | 2.23 | 0.0427 | 2.15 | 2.32 |
|  | Tree | native | 3.18 | 0.044 | 3.09 | 3.26 |
|  | Tree | non-native | 3.17 | 0.0422 | 3.09 | 3.26 |
| SLA | Shrub | native | 4.97 | 0.0254 | 4.92 | 5.02 |
|  | Shrub | non-native | 4.98 | 0.0209 | 4.93 | 5.02 |
|  | Tree | native | 5.03 | 0.0211 | 4.99 | 5.07 |
|  | Tree | non-native | 5.08 | 0.0207 | 5.04 | 5.13 |
| Totalphenol | Shrub | native | 1.59 | 0.0895 | 1.41 | 1.77 |
|  | Shrub | non-native | 1.74 | 0.0696 | 1.59 | 1.88 |
|  | Tree | native | 1.53 | 0.0704 | 1.39 | 1.68 |
|  | Tree | non-native | 1.29 | 0.0692 | 1.15 | 1.43 |
| Herbivory | Shrub | native | -4.24 | 0.0866 | -4.41 | -4.07 |
|  | Shrub | non-native | -4.43 | 0.0701 | -4.57 | -4.29 |
|  | Tree | native | -4.16 | 0.0712 | -4.3 | -4.02 |
|  | Tree | non-native | -4.07 | 0.0695 | -4.21 | -3.94 |

Note: Trait values were log-transformed (e.g., log(C), log(N), log(P), log(SLA)) or logit-transformed (logit(Herbivory+0.001)) before analysis. Means (emmean) and confidence intervals (lower.CL, upper.CL) are shown for native and non-native species within each life form (shrub, tree).
